# Clinical practice guidelines for the treatment of Ewing sarcoma (Spanish Sarcoma Research Group-GEIS)

**DOI:** 10.1007/s12094-024-03602-5

**Published:** 2024-08-19

**Authors:** Cristina Mata Fernández, Ana Sebio, Javier Orcajo Rincón, Javier Martín Broto, Antonio Martín Benlloch, David Marcilla Plaza, Antonio López Pousa, Isidro Gracia Alegría, Martina Giuppi, Erica Collado Ballesteros, Daniel Bernabeu, Enrique de Alava, Claudia Valverde Morales

**Affiliations:** 1 Pediatric and Adolescent Oncohaematology Unit, Hospital Materno-Infantil Gregorio, Marañón, Madrid, Spain; 2 Medical Oncology Department, Hospital Sant Pau, Barcelona, Spain; 3 Nuclear Medicine Department, Hospital General Universitario Gregorio Marañón, Madrid, Spain; 4 Medical Oncology Department, Fundación Jimenez Diaz University Hospital, University Hospital General de Villalba, and Instituto de Investigacion Sanitaria Fundacion Jimenez Diaz (IIS/FJD; UAM), Madrid, Spain; 5 Section Spine Unit. Orthopaedic and Traumatology Department, Dr. Peset University Hospital, Valencia, Spain; 6 Department of Pathology, Hospital Universitario Virgen del Rocío, Sevilla, Spain; 7 Orthopaedic Oncology Unit, Orthoapedic and Traumatology Department, Hospital Sant Pau, Barcelona, Spain; 8 Sarcoma patient, Madrid, Spain; 9 Radiation Oncologist, Hospital Universitari i Politécnic La Fe , Valencia, Spain; 10 Chief of Musculo-skeletal Radiology Section, Radiodiagnosis Service Hospital General Universitario La Paz, Madrid, Spain; 11 Department of Normal and Pathological Cytology and Histology, School of Medicine, University of Seville, Institute of Biomedicine of Sevilla, IBiS/Virgen del Rocio University Hospital /CSIC/University of Sevilla/CIBERONC, Seville, Spain; 12 Medical Oncology Department, Hospital Universitari Vall d´Hebrón, Barcelona, Spain

**Keywords:** Ewing sarcoma, Small round cell sarcomas, “Ewing like” sarcoma, Malignant bone tumours, EWSR1

## Abstract

Ewing sarcoma is a small round-cell sarcoma characterized by gene fusion involving EWSR1 (or another TET family protein like FUS) and an ETS family transcription factor. The estimated incidence of this rare bone tumor, which occurs most frequently in adolescents and young adults, is 0.3 per 100,000/year. Although only 25% of patients with Ewing sarcoma are diagnosed with metastatic disease, historical series show that this is a systemic disease. Patient management requires multimodal therapies—including intensive chemotherapy—in addition to local treatments (surgery and/or radiotherapy). In the recurrent/refractory disease setting, different approaches involving systemic treatments and local therapies are also recommended as well as patient inclusion in clinical trials whenever possible. Because of the complexity of Ewing sarcoma diagnosis and treatment, it should be carried out in specialized centers and treatment plans should be designed upfront by a multidisciplinary tumor board. These guidelines provide recommendations for diagnosis, staging, and multimodal treatment of Ewing sarcoma.

## Methodology

These guidelines were developed by a multidisciplinary panel of specialists from the different fields involved in the diagnosis and treatment of Ewing sarcoma (ES) in pediatric, adolescent, and adult patients. A bibliographic search was conducted for published articles in the PubMed database and also common guidelines were consulted, including those of the National Comprehensive Cancer Network (NCCN) [1], European Society for Medical Oncology (ESMO)/European Reference Network for Cancers (EURACAN) [2]. In several telematic consensus meetings, experts gave presentations for subsequent discussion by the multidisciplinary panel, which adopted the Infectious Disease Society of America levels of evidence/grades of recommendation (Table 1) [3].

**Table 1:** Levels of evidence and grades of recommendation (adapted from the Infectious Diseases Society of America-United Stated Public Health Service Grading System)

Levels of evidence	
I	Evidence from at least one large randomized, controlled trial of good methodological quality (low potential for bias) or meta-analyses of well conducted randomized trials without heterogeneity
II	Small randomized trials or large randomized trials with a suspiction of bias (lower methodological quality) or meta-analyses of such trials or trials with demonstrated heterogeneity
III	Prospective cohort studies
IV	Retrospective cohort studies or case–control studies
V	Studies without control group, case reports, expert opinions
Grades of recomendation	
A	Strong evidence for efficacy with a sustantial clinical benefit, strongly recommended
B	Strong or moderate evidence for efficay but with a limited clinical benefit, generally recommended
C	Insufficient evidence for efficacy or benefit does not outweight the risk or the disadvantages ( adverse events, costs,..) optional
D	Moderate evidence against efficay or for adverse outcome, generally not recommended
E	Strong evidence against efficacy or for adverse outcome, never recommended

## Introduction and epidemiology

These ES guidelines also cover the former entities known as Askin tumor and peripheral primitive neuroectodermal tumor (PNET). The ES group of small round-cell sarcomas are characterized by recurrent balanced translocations involving EWSR1 (or other TET family proteins like FUS) and an ETS family transcription factor. “Ewing-like” sarcomas or tumors lacking these characteristic fusions (including EWSR1/FUS non-ETS family members and CIC rearranged and BCOR—rearranged sarcomas), although currently similarly treated, have a different natural history and their management may differ, especially for CIC-rearranged tumors.

ES is the third most frequent malignant bone tumor in humans and the second most frequent after osteosarcoma in children. Median age at diagnosis is 15 years, and the male sex predominates (1.5:1) [4]. ES is practically non-existent in African and Afro-American populations, while the estimated incidence is 0.3 per 100,000/year in Caucasian adults.

Although the primary tumor is usually located in the bone (diaphysis of long bones like the femur, tibia or humerus pelvis, chest wall and spine), up to 10–20% of cases are extra-skeletal.

ES is a systemic disease. Although only 25% of patients have obvious metastases at diagnosis, in the past, 80–90% of patients with apparently localized disease died when treatment was reduced to a local approach. Disease dissemination is predominantly hematogenous, being lung, bones, and bone marrow as the most common metastatic sites.

## Clinical and radiological diagnosis

Persistent, localized bone pain (96% of cases), especially if asymmetric and causing the patient to wake at night, or a prolonged and unjustified limp, should raise suspicions and warrant further evaluation (evidence level grade: IV,A).

In conventional radiography, most ES appear as aggressive osteolytic lesions with a permeative pattern, most cases mixed lytic-sclerotic, or purely lytic. Around half of the lesions show complex periosteal reactions, such as multi- laminated “onion-skin”, “sunburst,” and Codman’s triangle. Also common is a non-calcified soft tissue mass extending around the bone and displacing adjacent fatty lines.

Computed tomography (CT) can help detect bone destruction, periosteal reaction, and soft tissue mass in anatomically complex regions like the pelvis, spine, and ankle. Sclerotic bone may be present in up to 40% of cases, mostly related to osteonecrosis in central medullar areas or in flat bones.

Magnetic resonance imaging (MRI) is very sensitive in detecting bone and soft tissue tumor components and in defining tumor relationship to nearby nerves, vessels and fascial planes) [5]. Because skip lesions can be expected, MRI protocols must include proximal and distal joints to the affected bone (V,A). ES shows a non-specific signal pattern: hypointense in T1-weighted imaging (WI) and slightly hyperintense in T2-WI. The soft-tissue mass is almost always connected through cortical breaks to the bone lesion; however, because of rapid infiltrative spread, some cases show no cortical disruption, and the soft component appears to be wrapped around the bone (the wraparound sign). Contrast enhancement (CE), in both CT and MRI, is usually strong and diffuse. Diffusion-MRI showing a clear restriction pattern with very low apparent diffusion coefficient (ADC) values can help differentiate edema or necrosis from tumor cellularity [6]. The imaging appearance of extra-skeletal ES is non-specific, as happens with any other soft-tissue sarcoma.

Differential diagnosis should include osteomyelitis, osteosarcoma, lymphoma, leukemia, Langerhans cell histiocytosis, and metastatic neuroblastoma.

Percutaneous core needle biopsy using the coaxial trocar system, is the standard outpatient procedure, although an incisional biopsy may be necessary in a small minority of patients [7] (III,A). Biopsy must be directed to selected areas with cellularity or metabolic activity, as detected by CE-MRI, Doppler ultrasound (US), diffusion-MRI or positron emission tomography PET/CT [8] (IV,A). It is recommended to obtain multiple samples from the same biopsy. In most referral centers, surgeons and musculoskeletal radiologists together evaluate the optimal biopsy path [9] to be removed during the final surgery (V,C).

## Pathology and molecular biology

Although gross examination of untreated ES specimens is now uncommon because of the standard use of neoadjuvant treatment, a cut surface is soft, and grey-white color, and frequently includes areas of hemorrhage and necrosis [10].

**Fig. 1:**
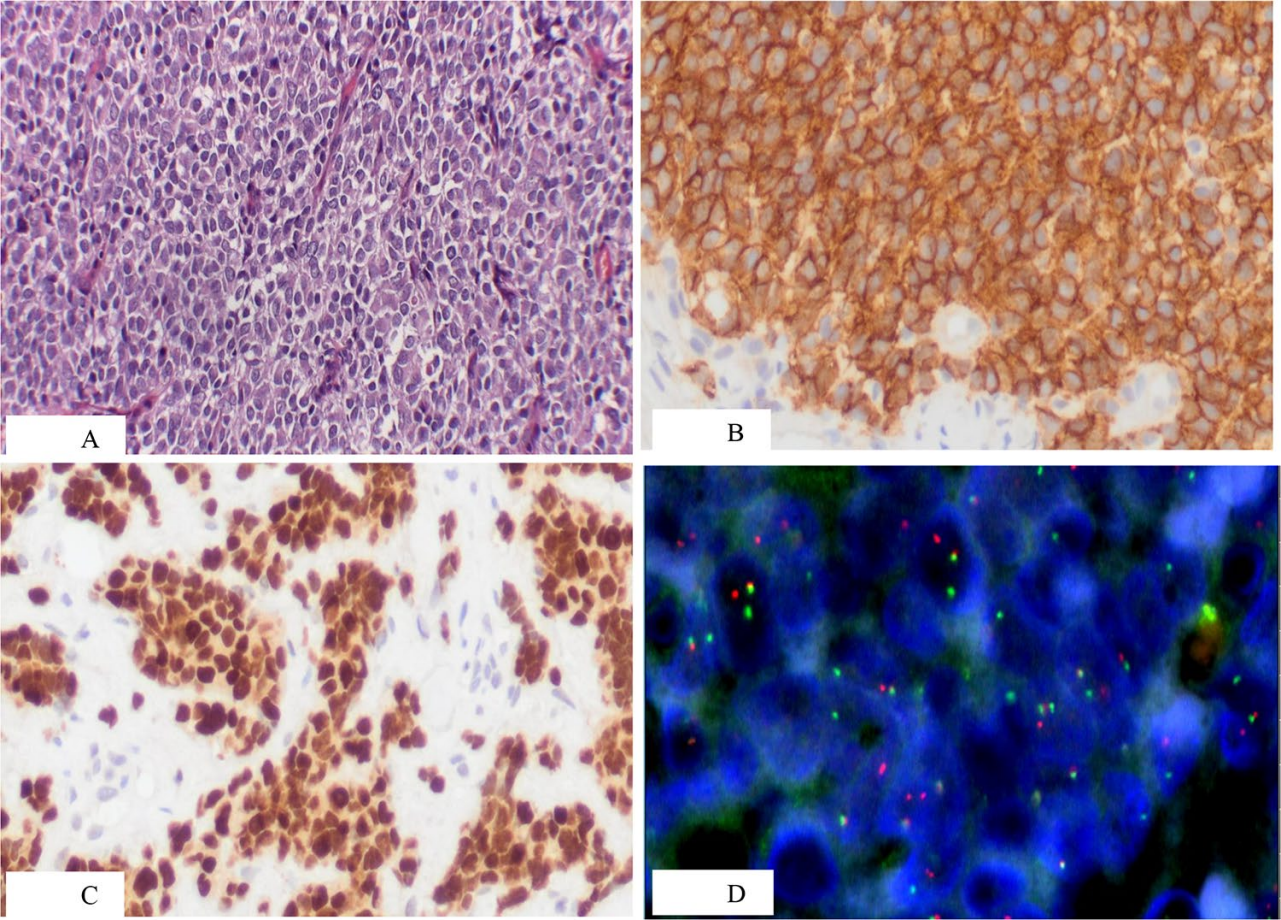
Hystologic and molecular pattern of Ewing sarcoma. **A** Ewing Sarcoma. Conventional appearance with uniform small round cell and scanty cytoplasm. **B** Ewing Sarcoma. Strong and diffuse membranous reactivity for CD99. **C** Ewing Sarcoma. Intense and diffuse nuclear expression of NKX2.2. **D** Ewing Sarcoma. FISH analysis showing rearrangement of EWSR1 (courtesy Dr. M. Biscuola)

Histologically, most cases are made up of solid sheets of uniform small round cells with round nuclei containing finely stippled chromatin, inconspicuous nucleoli, and scant clear or eosinophilic cytoplasm, with ill-defined cell borders [11] (Fig. 1A). Occasionally (in atypical ES), the tumor cells are larger, with more conspicuous nucleoli and irregular nuclear contours [12]. A minority of cases provide evidence of neuroectodermal differentiation with rosette formation.

Immunohistochemically, neoplastic cells show a membranous pattern of intense and diffuse CD99 expression (Fig. 1B) and NKX2.2 nuclear immunoreactivity (Fig. 1C). However, it is important to note that CD99 and NKX2.2 are not entirely specific for ES [13]. Nuclear FLI1 and ERG are often expressed in cases with the corresponding gene fusions [14].

Genetic confirmation is required for ES diagnosis. The most common ES translocations are t(11;22) (q24;q12), and. t(21;22) (q22;q12), resulting in the *EWSR1*-*FLI1* and *EWSR1*-*ERG* fusion transcripts and proteins (~ 85% and ~ 10% of cases, respectively); The remaining cases have alternative translocations that join *EWSR1* or *FUS* (which, along with *TAF15*, form the FET family) to other ETS family members. All cases of ES harbor a FET-ETS fusion. The most commonly used diagnostic approach is fluorescence in-situ hybridization (FISH) using an EWSR1 break-apart probe (Fig. 1D). Molecular techniques, such as reverse-transcriptase polymerase chain reaction (RT-PCR) which detects both translocation partners, are more specific for ES, while next- generation sequencing (NGS) techniques appear to be moving to the forefront of molecular diagnostic approaches.

The differential diagnosis includes tumors with small round-cell morphology, mainly round cell sarcoma with EWSR1 non-ETS fusion, CIC-rearranged sarcoma, and sarcoma with a BCOR genetic alteration.

Described as a distinct subset of lesions carrying the same fusions, predominantly in the head and neck region, is adamantinoma-like ES, which often expresses pan-cytokeratin and markers of squamous differentiation. However, the relationship of this tumor type to classic ES is uncertain [15].

Definitive ES diagnosis should be performed (or reviewed) at a reference sarcoma center and based on sufficient biopsy material for conventional histology, immunohistochemistry, and molecular pathology [16] (I,A).

## Staging and risk assessment

Staging is critical for treatment strategy planning and for prognostic assessment.

Initial laboratory investigations include blood count, serum biochemistry, and lactate dehydrogenase (LDH) testing.

CT scans show the highest sensitivity for lung metastasis detection. Extrapulmonary metastases may be ruled out by whole-body bone scans with 99mTc-hydroxydiphosphonate (HDP) bone scintigraphy (BS) or 18F-fluorodeoxyglucose FDG-PET/CT. Overall, PET/CT shows greater sensitivity (96%) and specificity (92%) for bone lytic metastasis detection [17] and it works especially well for nodal and soft tissue lesions.

Whole-body MRI may be an alternative to BS and PET/CT for bone marrow staging, especially when red bone marrow hyperplasia produces high tracer uptake [18]. Currently, most clinical guidelines recommend routine use of PET/CT for initial ES staging and surveillance (IV,A).

Bilateral iliac bone marrow biopsy is no longer standard in staging, because of the extremely low rate of bone marrow involvement in the absence of bone metastasis detection by BS or PET [19].

There is not standard staging classification system for ES and the American Joint Committee on Cancer (AJCC) bone cancer classification is not used by the most important international collaborative groups [20]. The most pragmatic approach to assigning a treatment strategy is therefore based on risk factors.

Systematic reviews on prognostic factors for ES 5-year overall survival (OS) conclude that independent prognostic factors on diagnosis are age (< 15 years vs ≥ 15 years) [21], volume (< 200 mL vs ≥ 200 mL) [22], primary tumor location (appendicular vs axial), and disease extent (local vs lung metastasis vs extra-pulmonary metastasis) [23], and, after neoadjuvant treatment, histological response poor vs good (< 10% of viable tumor cells) [24]. While other studied variables, such as LDH [25], surgical margins, and local treatment modalities have been reported as prognostic factors, they are not all widely accepted as such.

Based on the above prognostic factors, the following risk groups have been proposed: standard-risk or good-risk (localized disease < 200 mL and, good histological response), High or poor risk (localized disease ≥ 200 mL or, poor histological response) [26]. Patients with metastasis constitute the group with the poorest prognosis, and outcomes are better for lung metastasis than for extrapulmonary metastasis) [27].

## Treatment of localized disease on diagnosis

### Systemic treatment

It is currently surmised that most patients with localized ES will have subclinical metastasis on diagnosis. Therefore, the accepted standard treatment for localized ES is multimodal, based on intensive multiagent chemotherapy (CTh) in addition to local treatment. After complete assessment (hematological, liver, cardiac, and renal function, and fertility preservation when desired), induction CTh is initiated (for volume reduction and subclinical metastatic disease control), followed by local treatment, and finally by consolidation CTh. OS is 70% and 50% at 5 and 10 years, respectively.

Almost all active protocols are based on combinations of the 5–6 most active drugs (doxorubicin, cyclophosphamide, vincristine, etoposide, ifosfamide, and dactinomycin)*.*

Dose- dense regimens (every 2 weeks instead of the standard 3 weeks regimen) have been associated with a positive outcome in patients < 18 years [28]. The EuroEwing 2012 trial has also showed that an interval-compressed 2 weeks interval vincristine-doxorubicin- cyclophosphamide/ifosfamide-etoposide (VDC/IE) regimen is superior to a 3-week-interval vincristine-ifosfamide-doxorubicin-etoposide (VIDE) regimen [29] (I-A).

The consolidation use of high-dose (HD) CTh with stem-cell transplantation (SCT) for ES has been a topic of controversy in recent decades. A randomized study has demonstrated that, compared to seven cycles of maintenance chemotherapy, HD-CTh improves both event-free survival(EFS) and OS for patients aged < 50 years with localized high-risk ES (residual viable cells ≥ 10%) for patients undergoing surgery after induction chemotherapy alone or because of large tumor volume at diagnosis (≥ 200 mL) in unresected or initially resected tumors or resected tumors with preoperative irradiation but patients with a small unresected tumor were also eligible in case of poor clinical response to induction chemotherapy (< 50% radiologic reduction in soft tissue disease component); and no medical contraindication to treatment nor possibility of Busulfan interaction with radiotherapy [30, 31] (I,B).

Nevertheless, HD-CTh is not recommended for patients with metastasis to the pleura or to other sites, except within a clinical trial, as the benefit is unproven [32, 33]*.*

No specific clinical trials have been conducted with adults aged > 40 years. Several single-center series and a retrospective analysis of the Surveillance, Epidemiology and End Results (SEER) database show that survival rates approach pediatric survival rates when adults are treated with the same pediatric protocols [34–36] (II-B).

To summarize, our recommended approach for localized ES disease is based on EuroEwing 2012 Protocol (Table 2).

**Table 2:** First line treatment plan. Adapted from Euroewing 2012


Agents and Dosage
Induction 9 cycles given at 14 day intervals on haematological recovery (ANC ≥ 0.75 × 109/L, platelets ≥ 75 × 109/L, with G-CSF support after every cycle)
**VDC (1,5,9,13,17 weeks):** Vincristine: 2 mg/m^2^, d1 (IV push or short infusion)(max. single dose: 2 mg),, Doxorubicin 37.5 mg/m^2^/d (IV infusion, 24 h) d1, d2 (75 mg/m^2^/cycle)
Cyclophosphamide 1200 mg/m^2^ (IV infusion, 1 h) d1 plus MESNA and hydration*
**IE (3,7,11,15 weeks)** Ifosfamide 1800 mg/m2/d (IV nfusión, 1 h) d 1–5 (9 g/m^2^/cycle) plus MESNA and hydration*,Etoposide 100 mg/m^2^/d (IV nfusión, 2 h) d1–5 (500 mg/m^2^/cycle). (*^1^ according to institutional guidelines)
Consolidation
(**A) 5 cycles of alternating IE and VC at 14 day intervals** on haematological recovery, same requirements)
**IE (s.21,25,29)** as in induction regimen; **VC (s.23,27)** as an induction therapy. G-CSF support. Maximum dosis calculated for a SA of 2 m^2^
** (B) 1 cycle VAI plus BuMel:**
**VAI (s.21)** Vincristine 1.5 mg/m2 d1 (max. 2 mg), Actinomycin D 0.75 mg/m^2^/d (IV push) d1–2 (1.5 mg/m^2^/cycle) (max. single dose per day: 1.5 mg).Ifosfamide 3 g/m2/d (IV infusion, 1–3 h) d1–2 (6 g/m^2^/cycle) plus MESNA and hydration*
**BUMEL (s.23)** Busulfan, IV 2 h infusion, (total of 4 doses from day -6 to day-3: Adults and children > 34 kg: 3.2 mg/kg/24 h, Children and adolescents: > 23–34 kg 3,8 mg/kg/24 h; 16–23 kg; 4,4 mg/kg/24 h; 9– < 16 kg: 4,8 mg/kg/24 h; < 9 kg: 4 mg/kg/24 h; Melphalan 140 mg/m^2^ IV infusion over 30 min day -2 preinfusion. Hydration, G-CSF. Stem cell reinfusion on day 0. Clonazepam, and bone marrow transplantation support according to institutional guidelines
BuMel should be given on haematological recovery to ANC 1.0 × 10^9^/L, platelets 80 × 10^9^/L
Mobilization and Harvesting: Recommended following the 9th induction cycle VDC. An option would be to do it from a steady state with G-CSF 10 mcrgrs/kg/día from the -4 day to collect a total of at least 3 × 10^6^/kg CD34 + cells. G-CSF must be continued daily collection has been completed
(*)Contraindication to BuMel: BuMel HDCT may interact with radiotherapy and must not compromise the ability to deliver an effective radiotherapy dose. Specific techniques and contraindications must be individualized. Failed harvesting is clearly a contraindication as well as other individual factors that have to be considered: Advanced age, comorbidities…

### Response assessment

RECIST 1.1 is the standard oncology classification system for measuring response to treatment. Because ES bone lesions infrequently change in size, or show an asymmetric response to CTh, 3D-volumetric criteria [37] or reduced glycolytic activity outcomes [38] as evidenced by PET/CT, generally, correlate better with clinical

Pre- and post-treatment tumor glycolytic rate has been demonstrated to be a useful indirect measure of histological response to neoadjuvant CTh and progression-free survival (PFS). Recent studies have shown that metabolic response (> 55% maximum standardized uptake value (SUVmax) reduction) is associated with 80% 3-year EFS [39], evaluable as early as nine days after starting treatment [40]. Thus, a PET-/CT study is recommended for early neoadjuvant response assessment in ES (IV,A). Alternatives when PET-CT is not available are whole-body or local MRI with diffusion ADC, dynamic contrast enhancement and/or 3D-volume measures [41].

From the pathology perspective, response evaluation after neoadjuvant therapy, should be based on a full sagittal section and mapping of a whole representative slice of the tumor, as this allows evaluation of the relative proportions of viable and necrotic tumor [42].

### Surgery

Recommended local treatment options include wide excision, definitive RT, or, in selected cases, amputation [43]. However, surgery is the treatment of choice if tumor resection is possible with negative margins, without significant morbidity, and with a reasonable functional outcome.

Data from retrospective analyses suggest that surgery yields better local control and survival than definitive RT in patients with localized disease [44]. Selection bias, however, likely accounts for at least some of those reported results, since larger, axial lesions with a higher rate of local failure and overall poorer prognosis are more frequently referred for RT. It is also becoming clear that in less accessible locations, like the pelvis, preoperative RT and surgical resection are associated with better histological response and OS rates.

Surgery has several advantages over RT, including avoided risk of secondary radiation induced sarcomas, the possibility of knowing the tumor necrosis percentage after induction CTh, and that it avoids bone growth retardation and deformities associated with irradiation of the immature child skeleton.

Since options have not been directly compared in a randomized trial, the choice of local control treatments should be individualized, as it depends on tumor location, and size, response to CTh, anticipated morbidity, the patient’s age, and patient preferences. Patients and their treatment should be discussed by a multidisciplinary tumor board [45] (I,A).

Surgical management of patients with limb ES is a major challenge. The main objectives of current orthopedic oncology are optimal tumor resection and a functional extremity, with greater OS of both, the patient and the tumor reconstruction [46].

The surgical goal in ES is a wide-margin en-bloc resection (i.e., extirpation of the tumor with a normal tissue cuff covering margins) [47]. This usually means removing 2 cm of normal tissue (1 cm if an anatomical barrier is present), and performing a bone osteotomy at a distance of 3–5 cm from the bone involvement level (II,A). Smaller margins in the bone may be acceptable after effective neoadjuvant treatment. Bone resections with joint preservation that use open physis cartilage as a margin are also oncologically acceptable, as favoring preservation of the joint.

Amputation is indicated when, in limb sparing surgery, tumor resection with wide margins is not feasible, or when the outcome in terms of functionality is not acceptable.

Surgery should include removal of all tissues originally affected by the tumor, i.e., not just the tissues remaining after CTh shrinkage; when this is not possible, postoperative RT should be applied.

Regarding axial ES, there is no robust evidence in the literature for certain aspects of local management.

Neurological symptoms requiring laminectomy are common at presentation. However, in terms of neurological recovery, urgent decompressive surgery before an histological diagnosis is obtained may increase the risk of local recurrence, without providing a clear advantage over non-surgical treatment.

For patients with isolated (non-metastatic) spinal ES, surgical resection has been reported to be associated with significantly better OS than no surgery, after adjusting for age, RT, and extent of local tumor invasion [48, 49].

**Fig. 2:**
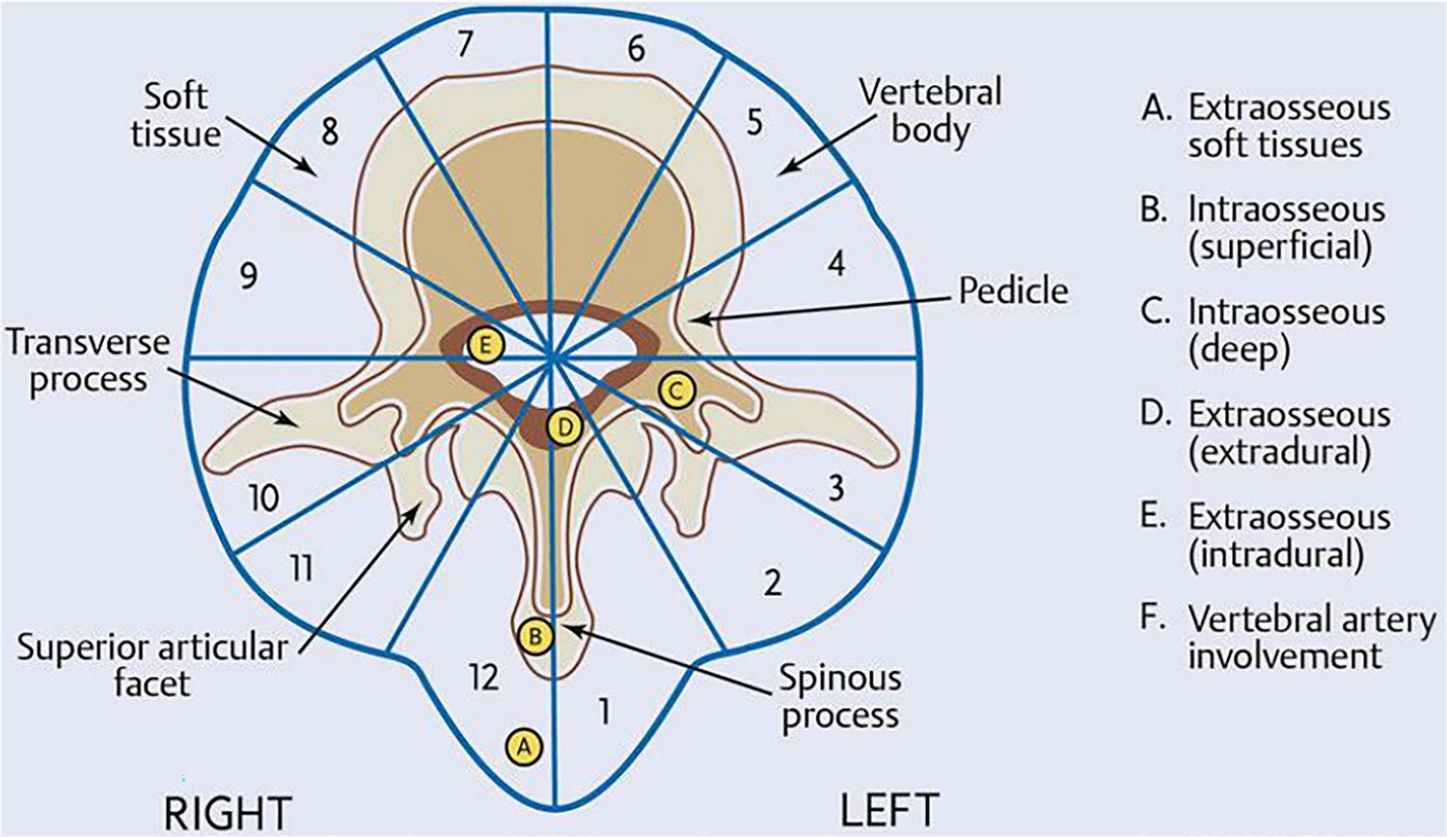
WBB (Weinstein, Boriani. Biagnini) Surgical Staging System. The transverse extension of the vertebral tumor is described with reference to 12 radiation zones (numbered 1 to 12 in a clokwise order) and to five concentric layers (**A**–**E** from the paravertebral extraosseus compartments to the dural involvement) The longitudinal extent of the tumor is recorded according to the levels involved

The additional challenge with axial tumors is that, at the moment of diagnosis, tumor margins have usually already exceeded the vertebral compartment with the involvement of paravertebral structures, and frequently, more than one vertebral segment. Thus, despite it is stated as wide-margin/ en- bloc -resection, spinal surgery is not radical, since the vertebral compartment must be opened to maintain the integrity of the uninvolved nervous structures [50] (Fig. 2).

As for implants, while carbon fiber-reinforced polyether etherketone (CFR-PEEK) and titanium implants have similar safety and efficacy profiles, CFR-PEEK has distinct advantages that make it a promising alternative for the treatment/outcome of spinal oncology patients, namely, that it decreases artefacts, improves early detection of local tumor recurrences, increases RT dose accuracy, and is also associated with lower complication rates.

For patients with primitive neuroectodermal tumor or extra-skeletal ES around the spine, the preferred treatment is multiagent CTh combined with wide- margin en-bloc excision and RT.

Finally, for unresectable spinal tumors, RT is the treatment of choice, as surgical debulking is not generally recommended.

### Radiotherapy

*RT as definitive local treatment* RT is an effective local control option for patients for whom function-preserving surgery is not possible because of tumor location or extent, and for patients with unresectable primary tumors despite induction CTh. In the latter case, debulking surgery should be avoided, so the treatment of choice is definitive radiotherapy [51] (II,A), using doses in the range of 54–60 Gy.

*RT as neo/adjuvant treatment* RT should be started within 60 days after surgery [52] (II,A) and should be considered in the following situations:

- Bulky tumors in difficult sites (pelvis, sacrum, spine, and paraspinal and rib tumors associated with pleural effusion), when RT can be preoperative (45 Gy) or postoperative.
- Residual microscopic tumors (R1: 45–50.4 Gy), gross disease after surgery (R2: 54–55.8 Gy), and inadequate surgical margins (i.e., marginal or intralesional surgery).
- High-risk chest wall primary tumors with close or involved margins, initial pleural effusion, pleural infiltration, or intraoperative contamination of the pleural space. For which adjuvant hemithorax irradiation doses typically range from 15 to 18 Gy (by age) plus a boost to the initial tumor.
- When histological response to preoperative CTh is poor (≤ 90%), even if surgical margins are negative (R0).

New RT technologies (such as stereotactic body RT (SBRT), intensity-modulated RT (IMRT), and proton therapy) potentially improve local control and toxicity outcomes. Recommended are conventional RT schedules of 1.8–2 Gy/fraction, once daily, rather than hyper-fractionated schedules.

Currently under evaluation are higher doses (64.8 Gy) for large tumors (> 8 cm, 100 mL) and lower doses (30–36 Gy) for small tumors (< 8 cm) that respond well to CTh.

## Treatment of metastatic disease on diagnosis

Although patients with metastasis on diagnosis have a significantly poorer prognosis (5-year OS 15–40%), treatment must be administered with curative intent, first, because it is difficult to predict which patients can be cured, and second, because treatment may relieve pain and prolong PFS [53].

### Systemic treatment

CTh regimens are broadly the same as for localized disease, except for certain considerations. Neither higher alkylator doses [54] nor HD-CTh with SCT [32, 33, 55] have shown any benefit for metastatic ES, so their use is not advisable except in clinical trials (I,C).

### Local treatment

Treatment is individualized according to the primary tumor, and the number and location of metastases.

*Primary site management* Local treatment of the primary tumor follows the same principles as for localized disease, but radiotherapy is preferred to amputation especially in patients with extensive metastatic disease.

*Pulmonary metastases* Since patients with few pulmonary metastases, have a better prognosis, they may be good candidates for surgical managements [56]. Repeated pulmonary metastasis excisions have been associated with improved OS [57].

SBRT of residual lung metastases is also a viable option [58, 59].

Retrospective reports from large cooperative groups and single center series suggest that bilateral whole-lung irradiation (WLI) benefits patients with pulmonary metastases, even when the metastases have been resected or have completely disappeared with CTh [60]. Long-term lung toxicity is acceptable [61], while patients with metastases who undergo surgery or boost RT have slightly higher complications.

Bilateral WLI is recommended at the end of systemic treatment for good responders, specifically, 15–18 Gy in daily doses of 1.8–2 Gy plus a focal boost to 40–50 Gy for gross residual metastases (II,B).

*Bone and soft tissue metastases* While patients with isolated bone or soft tissue metastases have a poorer prognosis than patients with isolated lung metastases, 10% may survive over the long term.

RT in doses of 40–50 Gy and SBRT for metastatic lesions are well tolerated and may improve disease control and symptoms [62] (IV,C).

Our treatment approach for EW with pulmonary metastasis is multimodal therapy including CTh, local treatment of the primary tumor and local treatment of the residual lesions (except in disseminated metastatic disease at the time of local treatment) (III, B). Treatment of residual disease may be followed up by low-dose WLI even when nodules have disappeared.

## Treatment on relapse

### Local relapse

Local relapse occurs in approximately 5% of patients undergoing limb rescue surgery for ES in specialized centers. Treatment of a local relapse depends on the time of recurrence, the association with distant metastases, and also resectability using the same criteria as for initial presentation. While prognosis for local ES relapse is usually poor, a non- significant impact on OS has also been reported [63].

RT and SBRT are options if the site has not previously been irradiated, and also as palliative care.

For local relapse, CTh is generally administered given the systemic nature of the disease and the high probability of developing metastasis if treatment is focused only on the local site (IV,B).

### Systemic relapse/progression

Prognosis for ES following systemic relapse is poor, with 5-year OS reported as 8–30% [64]. Most recurrences occur within the first 2 years after initial therapy and most patients develop metastatic disease or metastatic and local disease [65].

Time since initial therapy is the most important prognostic factor as prognosis is more favorable for patients whose relapse occurs more than 18 months after initial diagnosis.

Several CTh regimens have been used for relapsed ES, including alkylating agents, camptothecin derivatives, and platinum agents. Evidence regarding those regimens comes from small retrospective series or early trials with limited numbers of patients. In the last decade, four of the most frequently used CTh regimens have been High Dose ifosfamide (HD-IFO) [66], gemcitabine-docetaxel (Gem-Doc) [67], irinotecan-temozolomide (Irn-Tmz), and topotecan-cyclophosphamide (Topo-Cyc) [68] (II,B). An ongoing international European phase III trial, rEECur, comparing those four CTh regimens as treatment for refractory/recurrent ES, reported in a first interim analysis that the poorest objective response and PFS was obtained for Gem-Doc [69] and, in subsequent interim analyses, that Irn-Tmz and Topo-Cyc were both inferior to HD-IFO in terms of response, PFS, and OS [70] (I,A). The HD-IFO arm is currently being compared to carboplatin-etoposide and to Lenvatinib plus HD-IFO, and new arms will be sequentially added and compared.

While several retrospective studies have suggested a potential survival benefit for HD-CTh followed by SCT [71], as yet no randomized trial data support this as standard practice (IV,C). Tyrosine kinase inhibitors although not approved for ES, have been evaluated. The single-arm phase II CABONE study evaluated cabozantinib in 39 patients with ES, and reported an objective response of 26% and median PFS of months [72] (III,B), and the non-comparative, randomized, double-blind, placebo-controlled phase II study of regorafenib, reported a median PFS of 11.4 weeks [73].

Other novel CTh combinations under investigation include irinotecan plus trabectedin, which, in a recent phase I trial, showed encouraging activity and a good toxicity profile [74]. Finally, new molecules in monotherapy or combination therapy are currently under-evaluation in phase I trials.

## Follow-up

In the first 2 years after the end of treatment, local follow-up with clinical and physical examination every 2–3 months is usually enough for extremities. However, a structured local imaging protocol may benefit survival [75] (IV,B), and local magnetic resonance imaging (MRI) is recommended every quarter during the first two years (IV,B).

A metabolic study using FDG-PET/-CT, which has 92% sensitivity and 93% specificity for the detection of recurring bone sarcoma [76], is indicated in two main scenarios: when radiological images are inconclusive for local recurrence, and when prosthetic or osteosynthetic material is present in the patient [77] (III,A). Metallic components, however, do not cause significant artefacts that interfere with interpretation of FDG-PET-CT images.

Lung surveillance for metastases is usually by chest radiography or CT, and distant bone disease by PET/-CT or BS. For both bone and nodal surveillance, whole-body PET/-CT offers superior sensitivity, and is especially recommended in the case of new symptoms, abnormal imaging (primary tumors positive prior PET), and before any surgical decision-making [77] (IV,B). BS is reserved for cases when PET/-CT is not available (Table 3).

**Table 3:** Proposed Follow-up (Adapted from NCCN/BSG)

	First 2 years	3–5 years	> 5 years	> 10 years
Clinical&PE	Every 2–3 months	Every 4–6 months	Annually	Annually (II-B)
Local Imaging. MRI. Add US or PET if metal artifacts	Every 2–3 months	Every 4–6 months	Based on clinical indications or annually	Based on clinical indications or annually(II-B)
Chest Imaging Chest Rx or CT	Every 2–3 m (mind alternate CT & CRX)	Based on clinical indications or every 4-6 m (mind alternate CT & CRX)	Based on clinical indications or annually	Based on clinical indications or annually (II-B)
Body Imaging Whole-body PET/ CT or bone scan	Based on clinical indications or every 6 months	Based on clinical indications or annually	Based on clinical indications	Based on clinical indications

Note that the NCCN, ESMO and British Sarcoma Group (BSG) guidelines follow-up recommendations for high-grade sarcomas, including ES, vary (those in the BSG are less stringent). Recent evidence favors more individualized and less intensive follow-up regimes [78, 79].

## Patient-centered care

A radiological suspicion of malignant primary tumor in patients with a bone or soft tissue lesion should be referred to a networked sarcoma center with specialized and multidisciplinary expertise (IV,A). Children and adolescents, in particular, should be referred to centers with additional age-specific expertise.

Since ES occurs most frequently in children, adolescents, and young adults, communicating the diagnosis and its impact is particularly difficult. Psychological support for patients and family is highly recommended and clear communications between the patient and healthcare personnel is essential to avoid unfounded fears and concerns. Table 4 provides a short checklist for patients and physicians.

**Table 4:** Checklist for patients with ES

Patients should be attended at centers belonging to a sarcoma network with a concrete expertise and multidisciplinary team
Children and adolescents should be referred to centers which in addition provide age-specific expertise
A clear treatment plan with objectives and stimated timelines should be discussed with the patient
Psychological support for patients and families is highly recommended. Psychologists dedicated to childhood and adolescence can provide expert assistance
Basal assessment ( especially cardiological and endocrine) should be performed and other risk factors should be controlled in order to reduce toxicity burden of treatments
Oncofertility consultation should be assessed as soon as possible after the diagnosis
Encouraging of physical activity, adapted to patient situation, is highly recommended
Nutritional advice may give a greater sense of well-being and may help to control chemo and radio-therapy side effects
Basal work/ study activity should be assessed and patients should be refered to social workers as needed. Communication with school/university tutors and employers should be encouraged in order to facilitate a realistic plan for reintegration during and after treatment
Getting in touch with other patients and patients’ family through patient associations may reduce isolation feeling and should be offered
Quick activation of palliative care is essential (when indicated)
All patients are entitled to request a second opinion from other oncologist(s)/team

Treatment and follow-up visits should be scheduled taking into consideration quality of life, appropriate management of short- and long-term toxicities, early detection of relapses and secondary malignancies, and timely evaluation of co-morbidities and correlated risk patterns. Cardiological evaluation should include at least baseline and follow-up electrocardiogram (QTc interval and left ventricular ejection fraction).

Patient participation in clinical trials, although highly recommended, should be on an absolutely voluntary basis, and should be preceded by a family/patient discussion with the oncologist.

## Data Availability

Not applicable.
